# Supplementary material for: Prehistorical and historical declines in Caribbean coral reef accretion rates driven by loss of parrotfish
Source: Nat Commun. 2017 Jan 23;8:14160. doi: 10.1038/ncomms14160 (PMC5267576; doi:10.1038/ncomms14160)
Supplement: Supplementary Information — Supplementary Figures, Supplementary Tables and Supplementary References [file ncomms14160-s1.pdf]

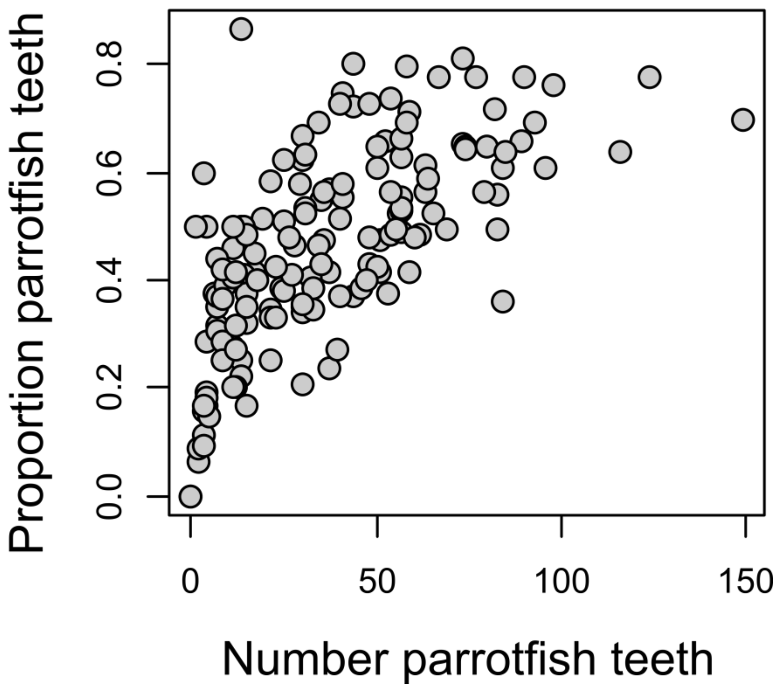

Supplementary Figure 1. Relationship between absolute and relative abundance of parrotfish teeth. Number samples = 154.

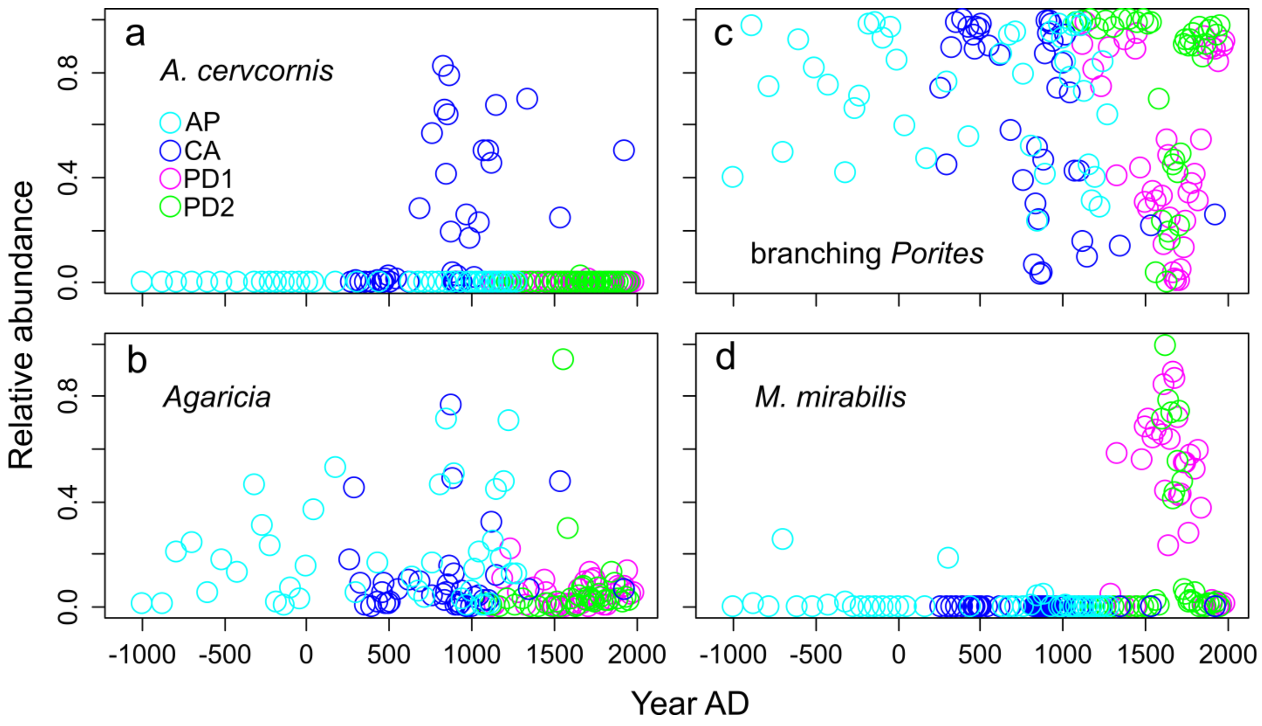

Supplementary Figure 2. Millennial-scale trends in abundance of common coral taxa. Abundance measured as weight of coral fragments of each taxon in > 2 mm sediment fraction divided by weight of all coral fragments in same fraction. (a) *Acropora cervicornis*. (b) *Agaricia* spp. (c) branching *Porites* spp. (d) *Madracis mirabilis*. Number samples = 154.

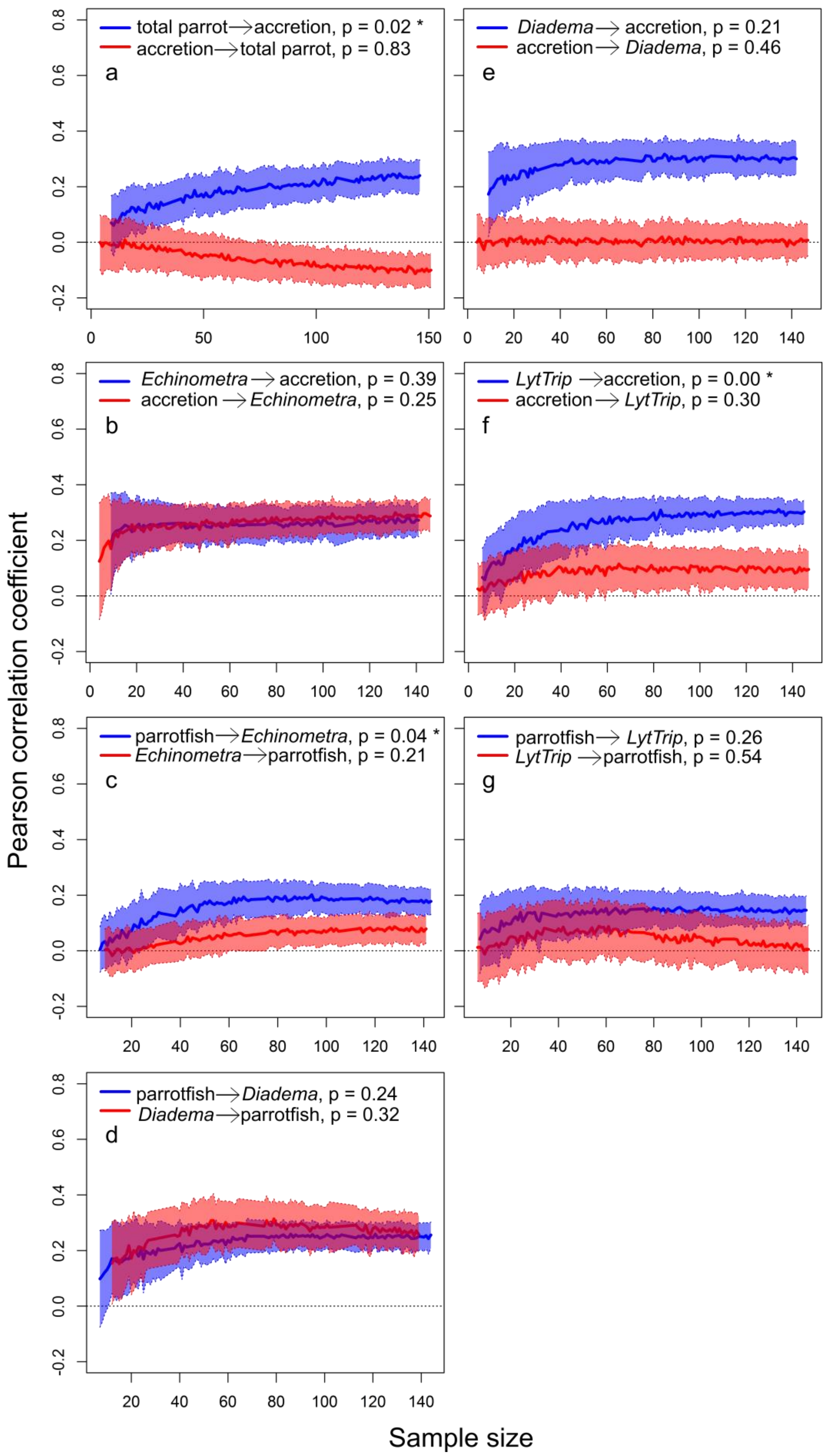
total parrot→accretion, p = 0.02 *
accretion→total parrot, p = 0.83
a
Diadema→ accretion, p = 0.21
accretion→ Diadema, p = 0.46
e
Echinometra→ accretion, p = 0.39
accretion → Echinometra, p = 0.25
b
LytTrip →accretion, p = 0.00 *
accretion→ LytTrip, p = 0.30
f
parrotfish→Echinometra, p = 0.04 *
Echinometra→parrotfish, p = 0.21
c
parrotfish→ LytTrip, p = 0.26
LytTrip →parrotfish, p = 0.54
g
parrotfish→Diadema, p = 0.24
Diadema→parrotfish, p = 0.32
d
Pearson correlation coefficient
Sample size

Supplementary Figure 3. Analysis of causality between reef accretion and abundance of herbivore taxa. (a) total parrotfish abundance and reef accretion rate. (b) *Echinometra* urchin abundance and reef accretion rate. (c) parrotfish relative abundance and *Echinometra* urchin abundance. (d) parrotfish relative abundance and *Diadema* urchin abundance. (e) *Diadema* urchin abundance and reef accretion rate. (f)) *Lytechinus* / *Tripneustes* urchin abundance and reef accretion rate. (g) parrotfish relative abundance and *Lytechinus* / *Tripneustes* urchin abundance. Lines and shaded regions show mean ± SD from 100 bootstrapped iterations. Significant causal forcing (*) determined from bootstrap test with 100 iterations, and indicated when the Pearson correlation coefficient is significantly greater than zero for larger sample sizes (number of core samples, including all spatial replicates in the composite time series) and when correlation coefficient increases significantly with increasing number of core samples.

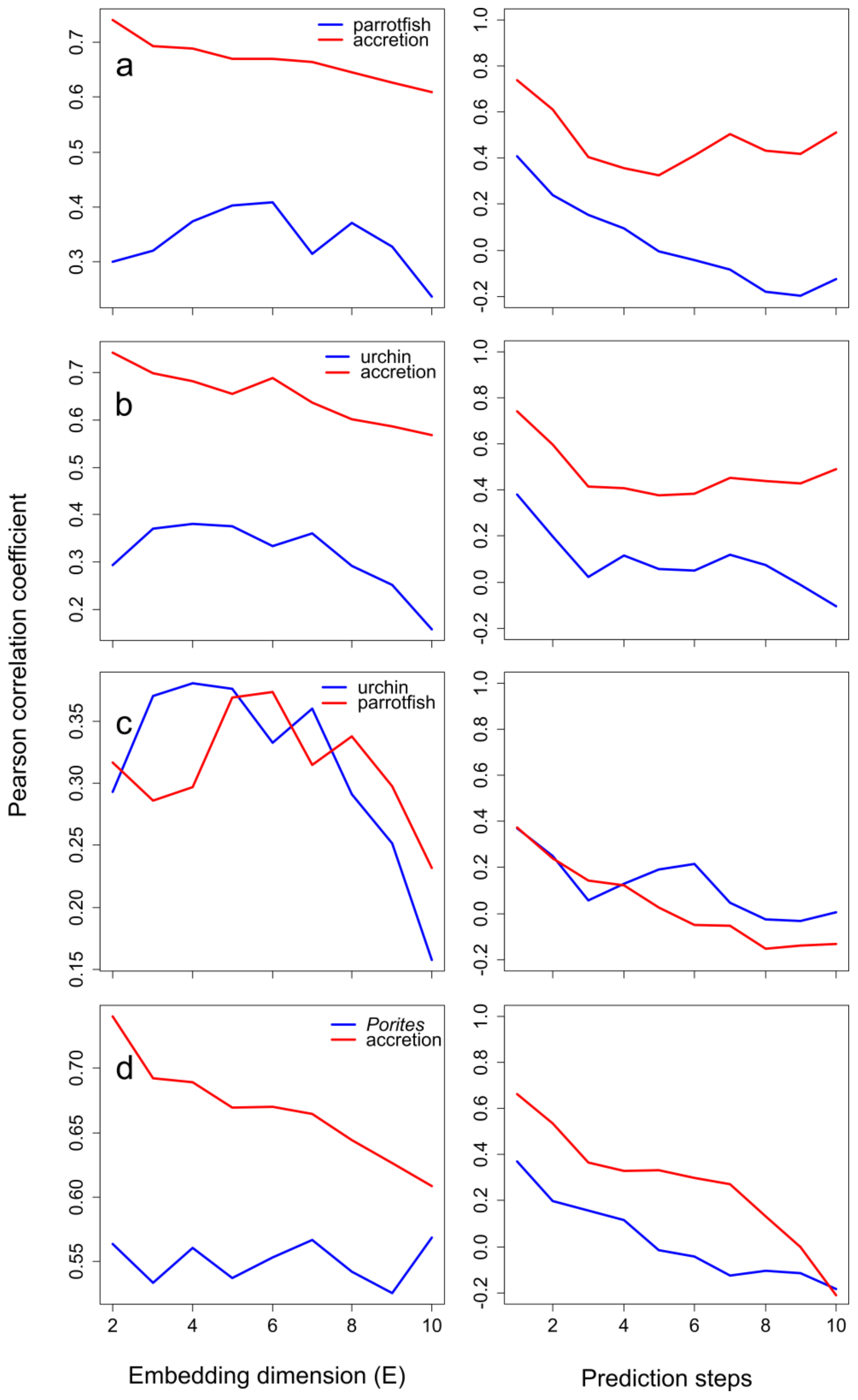

Supplementary Figure 4. Diagnostic plots for causality analyses shown in Figure 3. For each variable pairing, embedding dimension value that corresponded to highest Pearson correlation coefficient value was selected for analysis (left panel) and Pearson correlation coefficient was sufficiently high at short number of prediction steps and declined with increasing prediction time (right panel), confirming assumptions of nonlinearity and nonrandomness. (a) parrotfish relative abundance and reef accretion rate. (b) urchin abundance and reef accretion rate. (c) urchin abundance and parrotfish relative abundance. (d) branching *Porites* coral relative abundance and reef accretion rate.

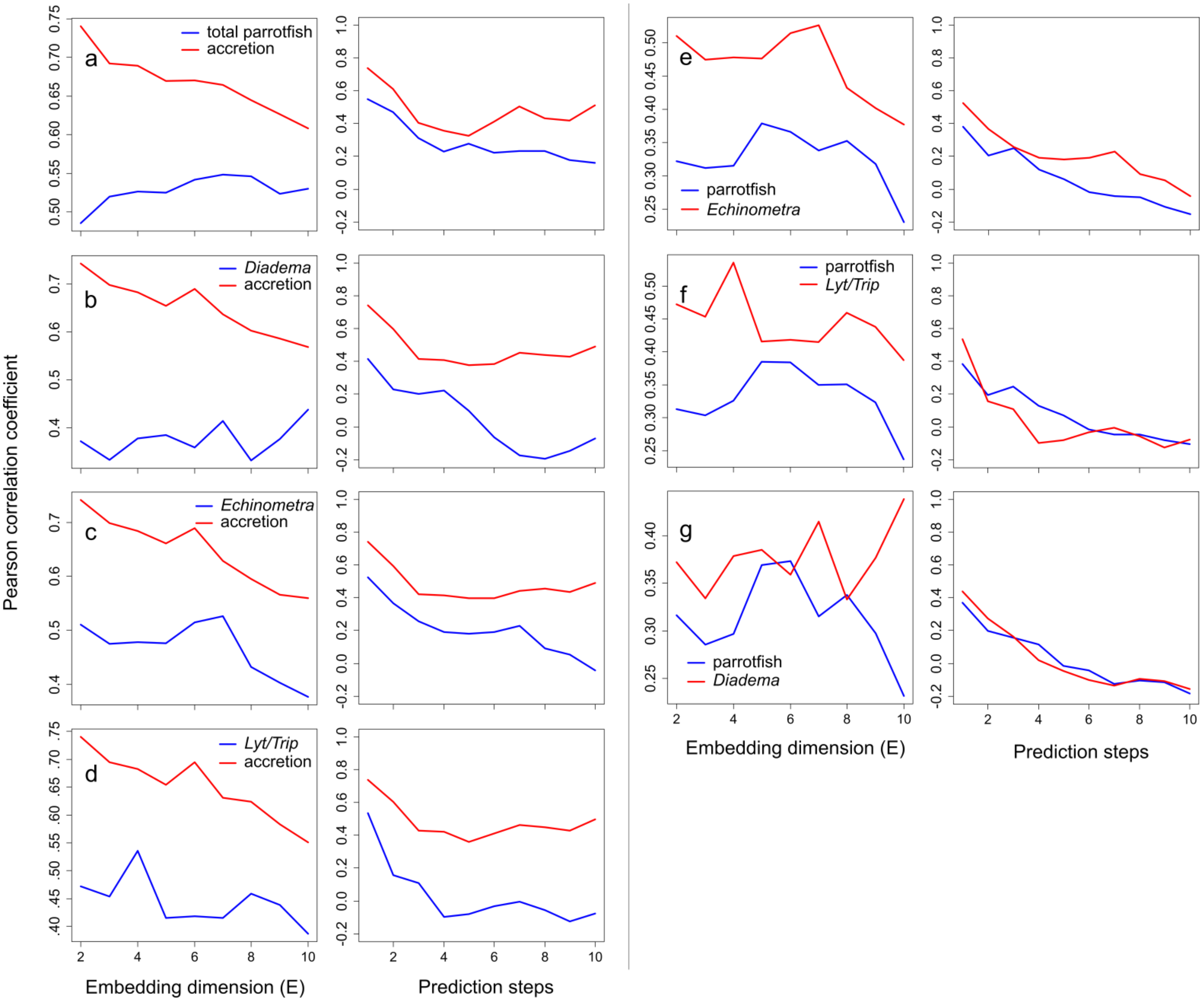

Supplementary Figure 5. Diagnostic plots for causality analyses shown in Supplementary Figure 3. For each variable pairing, embedding dimension value that corresponded to highest Pearson correlation coefficient value was selected for analysis (left panel) and Pearson correlation coefficient was sufficiently high at short number of prediction steps and declined with increasing prediction time (right panel), confirming assumptions of nonlinearity and nonrandomness. (a) parrotfish total abundance and reef accretion rate. (b) *Diadema* urchin abundance and reef accretion rate. (c) *Echinometra* urchin abundance and reef accretion rate. (d) *Lytechinus/Tripneustes* urchin abundance and reef accretion rate. (e) parrotfish relative abundance and *Echinometra* urchin abundance. (f) parrotfish relative abundance and *Lytechinus/Tripneustes* urchin abundance. (g) parrotfish relative abundance and *Diadema* urchin abundance.

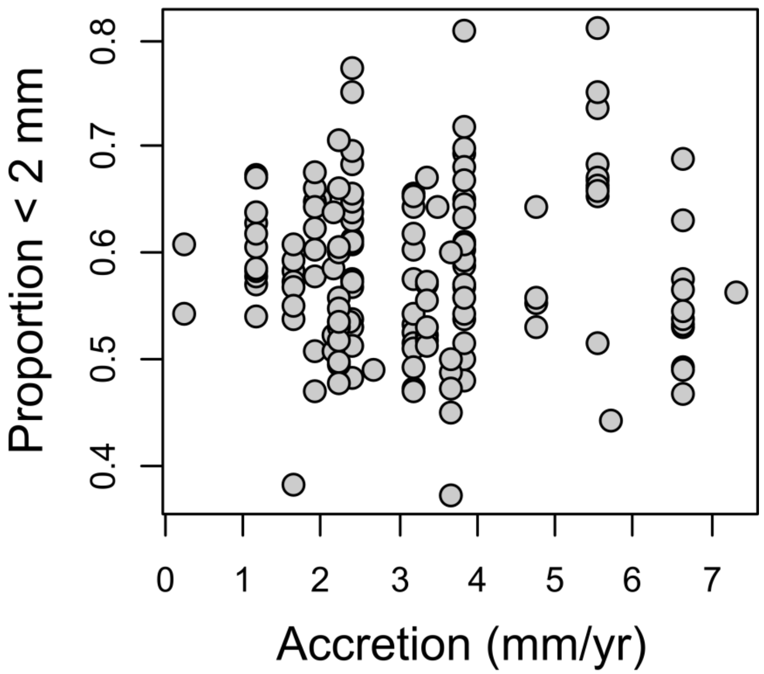

Supplementary Figure 6. Relationship between reef accretion rate and proportion of sample weight composed of smaller grains < 2mm. Number samples = 154.

Supplementary Table 1. MC-ICP-MS ^{230}Th ages of coral fragments obtained from reef matrix cores collected in the Caribbean. Two cores were obtained per site: one core was "well-dated" (with 6-8 ages per core) while the second "replicate" core was constrained by only a top and bottom age.

Core	Core positon (mm from top)	Sample weight (g)	U (ppm)	^{232}Th (ppb)	(^{230}Th/^{232}Th)	(^{230}Th/^{238}U)	[a]δ^{234}U	Time of chemistry (AD)	Uncorr. ^{230}Th age (BC/AD)	[b]Corr. ^{230}Th age (BC/AD)	[c]Corr. ^{230}Th age (BP)
Airport Point	0	0.15459	2.9751 ± 0.0015	2.357 ± 0.056	30.26 ± 0.74	0.007902 ± 0.000048	146.0 ± 1.3	2014.83	1260.4 ± 4.7	1280 ± 6	669.7 ± 6.2
	225	0.15552	3.0049 ± 0.0012	0.794 ± 0.027	88.8 ± 3.1	0.007728 ± 0.000072	147.0 ± 1.2	2014.83	1277.8 ± 6.9	1287 ± 7	662.5 ± 7.2
	425	0.15160	2.8445 ± 0.0012	2.93 ± 0.31	32.7 ± 3.4	0.011069 ± 0.000049	147.2 ± 1.0	2014.83	957.8 ± 4.8	982 ± 7	967.6 ± 7.3
	675	0.15133	2.8099 ± 0.0013	0.654 ± 0.025	142.6 ± 5.5	0.010941 ± 0.000055	145.2 ± 1.1	2014.83	968.3 ± 5.4	978 ± 6	972.3 ± 5.8
	1755	0.15369	2.7791 ± 0.0013	2.18 ± 0.11	80.4 ± 4.0	0.02077 ± 0.00010	147.7 ± 1.4	2014.83	24 ± 10	44 ± 11	1906 ± 11
	2960	0.17689	2.8705 ± 0.0012	4.04 ± 0.25	52.9 ± 3.3	0.024566 ± 0.000083	146.5 ± 1.3	2014.83	-345.9 ± 8.5	-314 ± 11	2264 ± 11
	4085	0.16048	2.9114 ± 0.0013	0.788 ± 0.039	352 ± 18	0.03135 ± 0.00013	146.4 ± 1.1	2014.83	-1007 ± 13	-997 ± 13	2947 ± 13
Airport Point (replicate)	0	0.19340	2.7590 ± 0.0014	2.075 ± 0.066	43.76 ± 1.41	0.010846 ± 0.000067	149.6 ± 1.0	2014.83	981.4 ± 6.4	1001 ± 8	949.2 ± 7.5
	3200	0.15195	2.9495 ± 0.0015	4.11 ± 0.14	65.44 ± 2.25	0.03002 ± 0.00011	146.3 ± 0.9	2014.83	-877 ± 11	-846 ± 12	2796 ± 12
Cayo Adriana	0	0.30314	2.6480 ± 0.0010	1.7964 ± 0.0022	4.995 ± 0.075	0.001117 ± 0.000017	148.4 ± 0.9	2013.89	1907.7 ± 1.6	1925.9 ± 4.0	24.1 ± 4.0
	200	0.15626	3.08156 ± 0.00090	0.10792 ± 0.00059	788.46 ± 7.32	0.009100 ± 0.000068	147.4 ± 0.7	2014.83	1146.7 ± 6.5	1152 ± 7	798.1 ± 6.6
	400	0.16861	2.6144 ± 0.0011	2.6300 ± 0.0033	37.50 ± 0.19	0.012432 ± 0.000062	147.5 ± 0.9	2014.83	827.2 ± 6.0	852 ± 8	1098.2 ± 7.8
	600	0.28834	2.56942 ± 0.00064	1.1296 ± 0.0011	78.38 ± 0.35	0.011357 ± 0.000050	147.1 ± 0.6	2013.89	927.6 ± 4.9	941.5 ± 5.6	1008.5 ± 5.6
	1225	0.12145	2.9608 ± 0.0024	0.40842 ± 0.00054	273.83 ± 2.54	0.01245 ± 0.00011	146.5 ± 1.2	2013.89	822 ± 11	829.4 ± 11.2	1121 ± 11
	1825	0.23607	2.73034 ± 0.00071	0.9260 ± 0.0011	137.71 ± 0.55	0.015392 ± 0.000059	146.9 ± 0.6	2013.89	538.9 ± 5.7	550.5 ± 6.2	1399.5 ± 6.2
	2410	0.22407	2.57093 ± 0.00069	4.9181 ± 0.0037	27.54 ± 0.12	0.017364 ± 0.000074	146.8 ± 0.6	2013.89	348.3 ± 7.2	390.4 ± 11.1	1560 ± 11
	3010	0.15027	2.6653 ± 0.0012	2.4439 ± 0.0030	61.18 ± 0.31	0.018489 ± 0.000092	147.6 ± 1.2	2013.88	240.8 ± 9.1	263.6 ± 10.2	1686 ± 10
Cayo Adriana (replicate)	0	0.15520	3.4257 ± 0.0016	1.2440 ± 0.0027	76.92 ± 0.44	0.009205 ± 0.000049	145.6 ± 1.3	2014.83	1135.2 ± 4.8	1146 ± 5	803.7 ± 5.3
	4855	0.18861	3.5112 ± 0.0023	2.5673 ± 0.0032	140.59 ± 0.60	0.03388 ± 0.00014	145.4 ± 1.1	2014.83	-1257 ± 14	-1239 ± 15	3189 ± 15

Core	Core positon (mm from top)	Sample weight (g)	U (ppm)	^{232}Th (ppb)	(^{230}Th/^{232}Th)	(^{230}Th/^{238}U)	[a]δ^{234}U	Time of chemistry (AD)	Uncorr. ^{230}Th age (BC/AD)	[b]Corr. ^{230}Th age (BC/AD)	[c]Corr. ^{230}Th age (BP)
Punta Donato 1	0	0.23014	2.58328 ± 0.00083	0.9665 ± 0.0013	3.610 ± 0.089	0.000445 ± 0.000011	146.8 ± 1.0	2013.89	1971.5 ± 1.0	1984.1 ± 2.8	-34.1 ± 2.8
	535	0.18008	2.8251 ± 0.0015	1.647 ± 0.062	14.77 ± 0.57	0.002838 ± 0.000023	146.7 ± 1.1	2014.83	1744.6 ± 2.2	1761 ± 4	189.3 ± 4.0
	1135	0.12697	2.94011 ± 0.00087	0.86495 ± 0.00090	45.45 ± 0.46	0.004406 ± 0.000045	147.8 ± 0.8	2013.89	1593.9 ± 4.3	1604.3 ± 4.8	345.7 ± 4.8
	2185	0.19065	2.55917 ± 0.00058	0.69644 ± 0.00092	67.56 ± 0.55	0.006059 ± 0.000049	147.2 ± 0.6	2013.89	1435.7 ± 4.7	1446.4 ± 5.2	503.6 ± 5.2
	3140	0.16530	2.77901 ± 0.00079	0.90693 ± 0.00093	85.65 ± 0.67	0.009212 ± 0.000071	147.5 ± 0.8	2013.89	1133.9 ± 6.9	1145.2 ± 7.2	804.8 ± 7.2
	3235	0.15000	2.9921 ± 0.0016	3.1078 ± 0.0060	28.65 ± 0.19	0.009806 ± 0.000063	146.6 ± 1.1	2013.88	1076.1 ± 6.1	1100.7 ± 7.9	849.3 ± 7.9
Punta Donato 2	0	0.16196	2.7810 ± 0.0016	1.6070 ± 0.0014	4.125 ± 0.069	0.000786 ± 0.000013	148.3 ± 1.0	2014.83	1940.2 ± 1.3	1956 ± 3	-6.3 ± 3.5
	3245	0.15443	2.7268 ± 0.0015	2.8458 ± 0.0029	34.74 ± 0.22	0.011949 ± 0.000076	146.4 ± 1.0	2014.83	872.5 ± 7.4	898 ± 9	1052.4 ± 8.9

Ratios in parentheses are activity ratios calculated from atomic ratios using decay constants of ref. 1. All values have been corrected for laboratory procedural blanks. All errors reported as 2-sigma (2σ). Uncorrected ^{230}Th ages (BC/AD) were calculated using Isoplot/EX 3.0 program[2], where BC = Before Christ and AD = Anno Domini.

[a]$\delta^{234}U = [(^{234}U/^{238}U)-1] \times 1000$.

[b]^{230}Th ages were corrected using a modelled two-component correction value based on the equation in ref 3. where the $^{232}Th_{dead}$ value used was the measured ^{232}Th value (ppb) in the non-living coral sample of this study. $^{232}Th_{live}$ was assumed to approximate the mean measured ^{232}Th value (ppb) of living coral specimens (0.95 ppb) found on the GBR (Great Barrier Reef) as no local values have been obtained for the Caribbean. Similarly, the $^{230}Th/^{232}Th_{live}$ value represents or approximates the isotopic composition of the hydrogenous component in the dead coral skeleton with an activity value of 1.08 ± 20% determined from live corals of the GBR. $^{230}Th/^{232}Th_{sed}$ is the detrital component represented by a by a mean activity value of 0.61 ± 20% from isochron derived initial ^{230}Th/^{232}Th values obtained from dead *Porites* coral skeletons of the GBR.

[c]Corrected ^{230}Th age in years before present (BP; where 'present' is 1950 AD).

Supplementary References

1. Cheng, H., Edwards, R.L., Hoff, J., Gallup, C.D., Richards, D.A., Asmerom, Y. (2000). The half-lives of uranium-234 and thorium-230. *Chem. Geol.*, 169, 17e33.
2. Ludwig, K.R., 2003b. Users Manual for Isoplot/Ex Version 3.0: a Geochronological Toolkit for Microsoft Excel. Berkeley Geochronology Centre, Berkeley. Special Publication No.3.
3. Clark, T.R., Roff, G., Zhao, J., Feng, Y., Done, T.J., Pandolfi, J.M. (2014). Testing the precision and accuracy of the U-Th chronometer for dating coral mortality events in the last 100 years. *Quat. Geochronol.*, 23, 35-45.
